# Supplementary material for: Multiple convergent supergene evolution events in mating-type chromosomes
Source: Nat Commun. 2018 May 21;9:2000. doi: 10.1038/s41467-018-04380-9 (PMC5962589; doi:10.1038/s41467-018-04380-9)
Supplement: Supplementary file 1 — Supplementary Information [file 41467_2018_4380_MOESM1_ESM.pdf]

Supplementary Information

Multiple convergent supergene evolution events in mating-type chromosomes

Branco et al.

Supplementary Information Includes:

Supplementary Figures 1 – 10
Supplementary Tables 1 – 6
Supplementary Note 1
Supplementary References

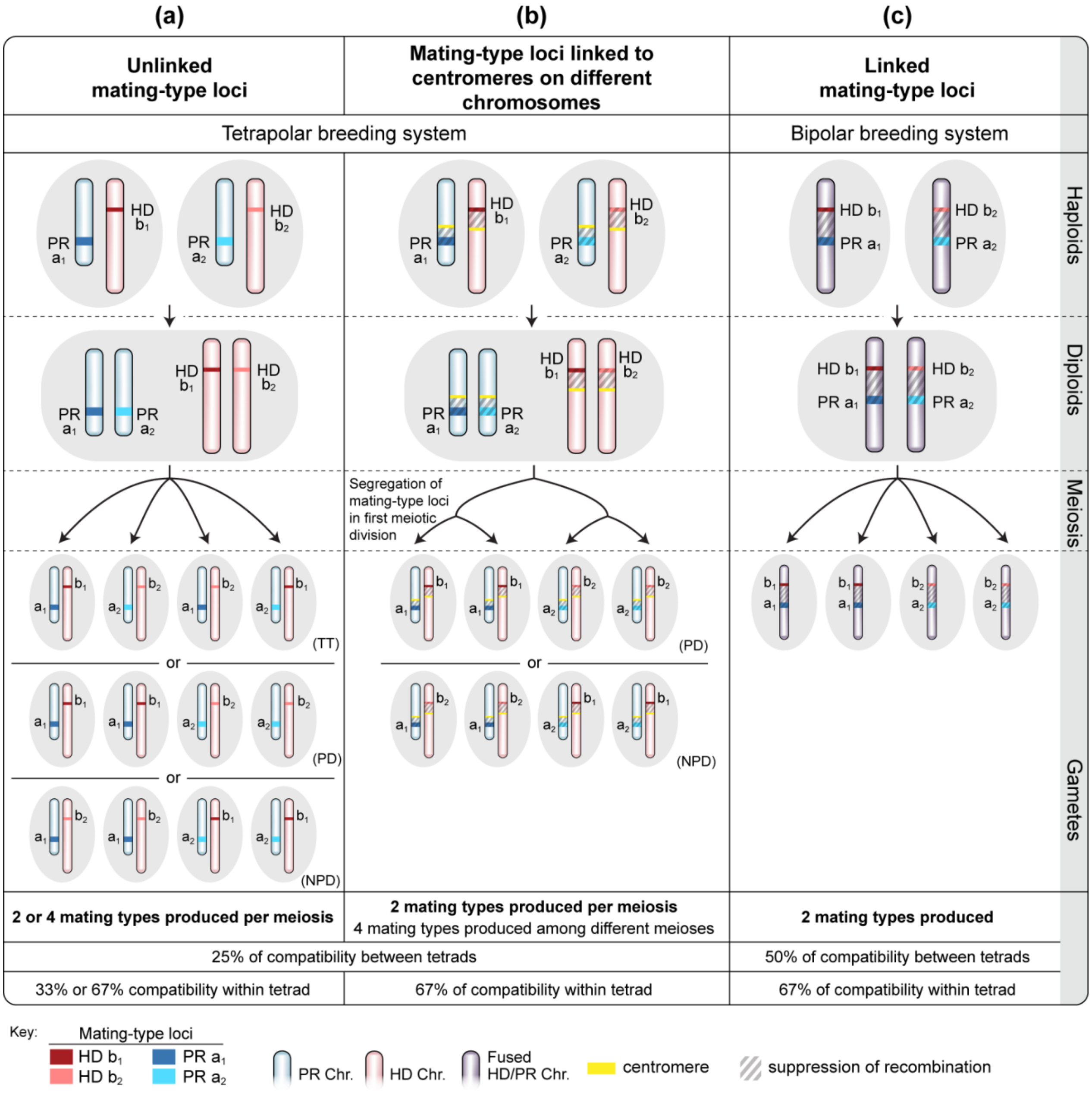

Supplementary Figure 1. Odds of compatibility among gametes of a diploid individual in basidiomycete fungi. In basidiomycetes gametes are fully compatible only if they carry different alleles at both mating-type loci, i.e., the *PR* (including pheromone receptor and pheromone genes, with a1 and a2 alleles) and *HD* (including homeodomain genes, with b1 and b2 alleles) loci. **(a)** when the *PR* and *HD* mating-type loci are completely unlinked (with no mating-type loci or centromere linkage and loci located on different chromosomes, depicted in blue and red, respectively), the percentage of compatibility of a given gamete with the other gametes produced by the same diploid individual is 25% across multiple meioses (a given gamete is compatible with one of every four gametes), and the percentage within tetrad is 33% (a given gamete is compatible with one of the other three gametes in the tetrad) or 67% (a given gamete is compatible with two of the three remaining gametes in the tetrad) depending on segregation of the mating type alleles. The different types of gametes produced are tetratypes (TT), parental ditypes (PD) or non-parental ditypes (NPD), that result from allele segregation and whether crossing-over occurs between one of the two loci and the centromere; **(b)** when the *PR* and *HD* mating-type genes are linked to the centromeres of different chromosomes (blue and red, respectively), the percentage of compatibility of a given gamete with the other gametes produced by the same diploid individual is 25% across multiple meioses but 67% within a tetrad (a given gamete is compatible with two of the three other gametes in the tetrad) due to the segregation of variation occurring only at meiosis I for both mating type loci. The different types of gametes produced are parental ditypes (PD) or non-parental ditypes (NPD), which depend on segregation; **(c)** when the *HD* and *PR* loci are fully linked to each other on the same chromosome, the percentage of compatibility of a given gamete among the other gametes produced by the same diploid individual is 50% across multiple meioses (a given gamete is compatible with one of every two gametes), and 67% within a single meiotic tetrad.

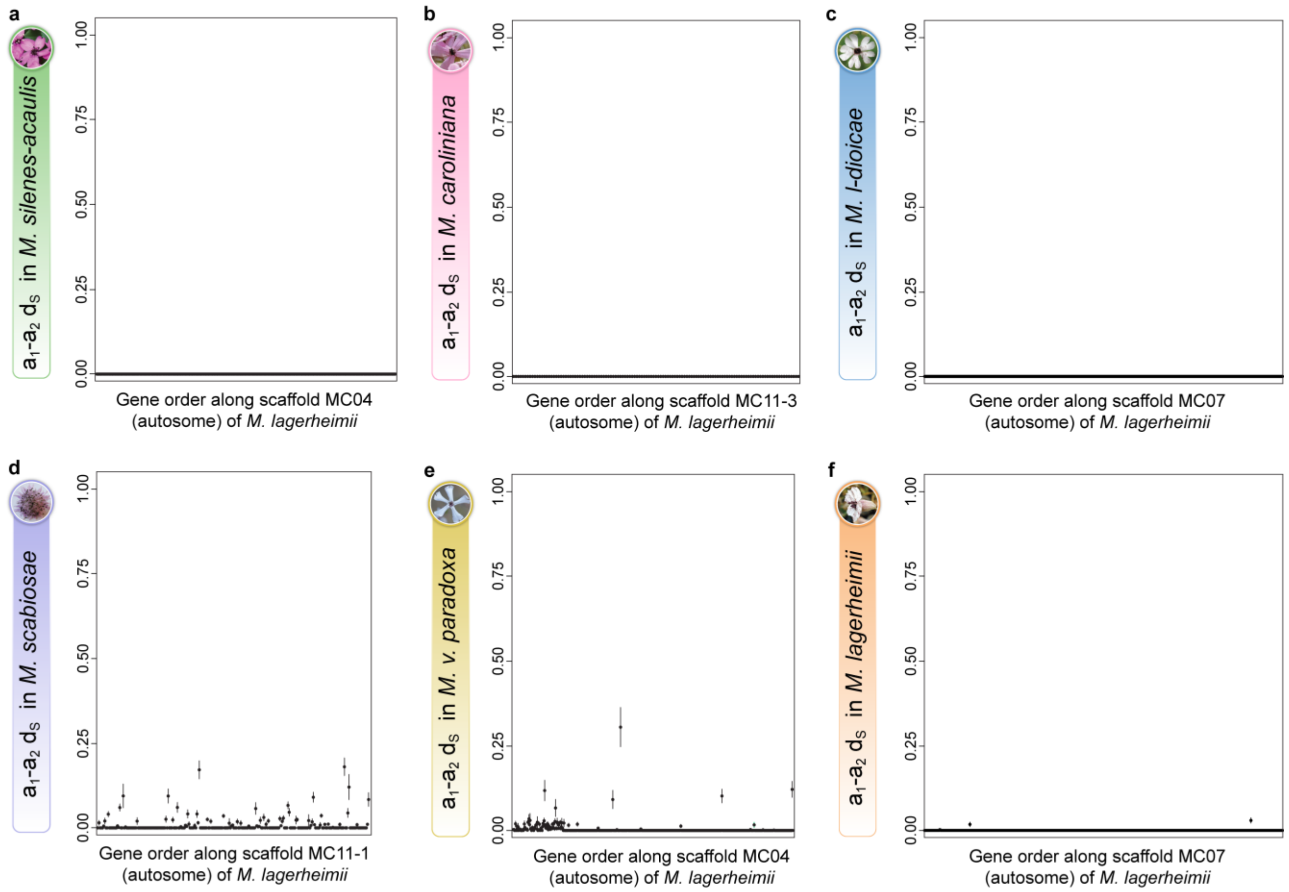

Supplementary Figure 2. Per-gene synonymous divergence and standard error (d_S ± SE) between alleles in the a_1 versus a_2 haploid genomes of an autosome per species, following the gene order of the homologous *Microbotryum lagerheimii* autosomes. Standard errors were estimated with the yn00 program of the PAML package. We selected a representative, well assembled, autosome for each species (number of genes N and contig lengths L are indicated in brackets): **(a)** *M. silenes-acaulis* (N=539 genes, L=1821 kb); **(b)** *M. v. caroliniana* (N=120 genes, L=481 kb); **(c)** *M. lychnidis-dioicae* (N=499 genes, L= 1585 kb); **(d)** *M. scabiosae* (N=235 genes, L= 1030 kb); The relatively high d_S results from the sequencing of haploid genomes from different individuals; **(e)** *M. v. paradoxa* (N=533 genes, L= 1822 kb); The relatively high d_S in one part of autosomes likely results from an outcrossing event followed by a selfing event; **(f)** *M. lagerheimii* (N=547 genes, L= 1585 kb).

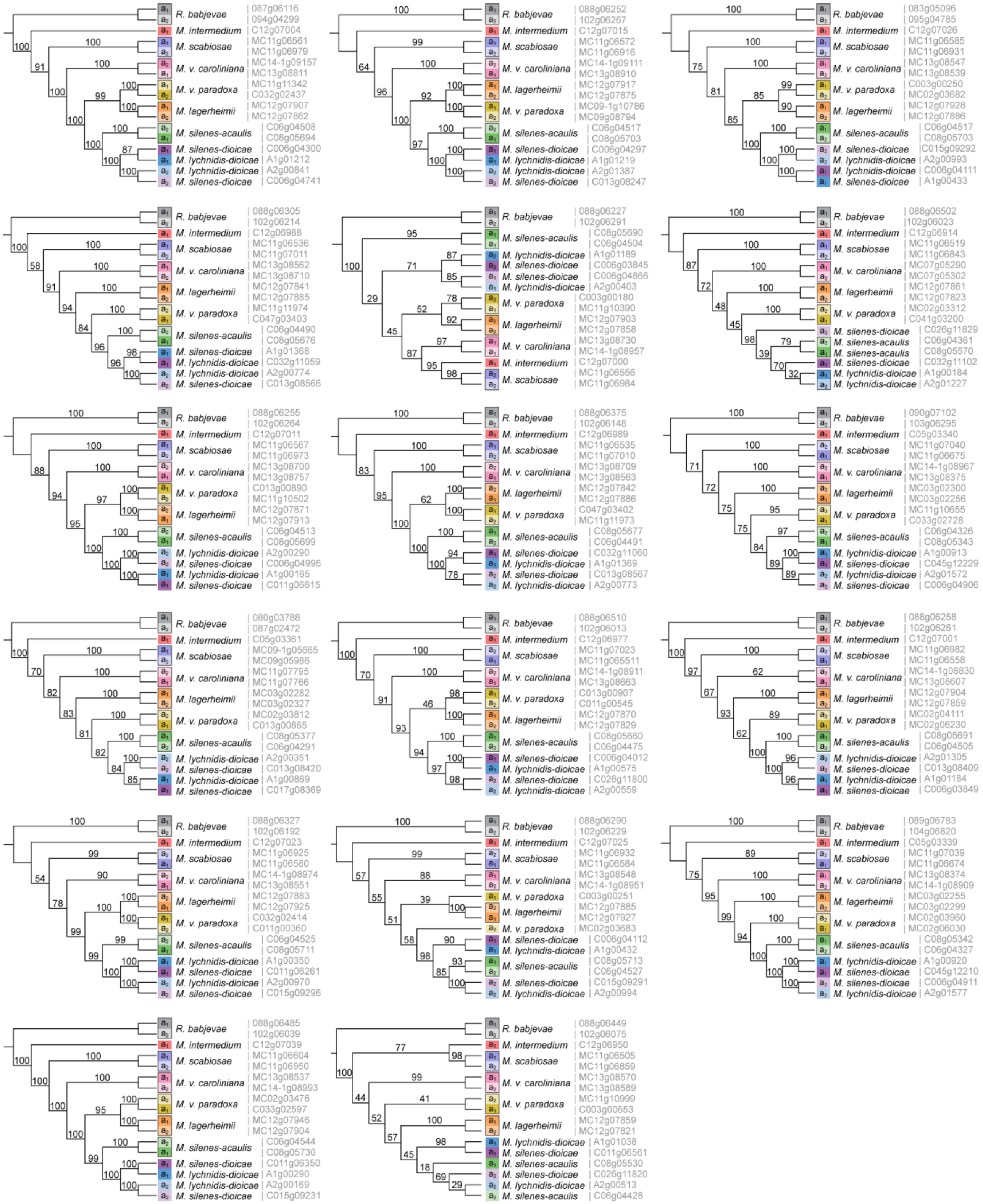

Supplementary Figure 3. Individual genealogies for the 17 genes used for dating the linkage of mating-type loci. The individual genealogies of these 17 genes ancestrally located between the *HD* and *PR* loci illustrate that trans-specific polymorphism in this genomic region occurs only between *Microbotryum lychnidis-dioicae* and *M. silenes-dioicae*, supporting that linkage between the two mating-type loci occurred independently in the other species.

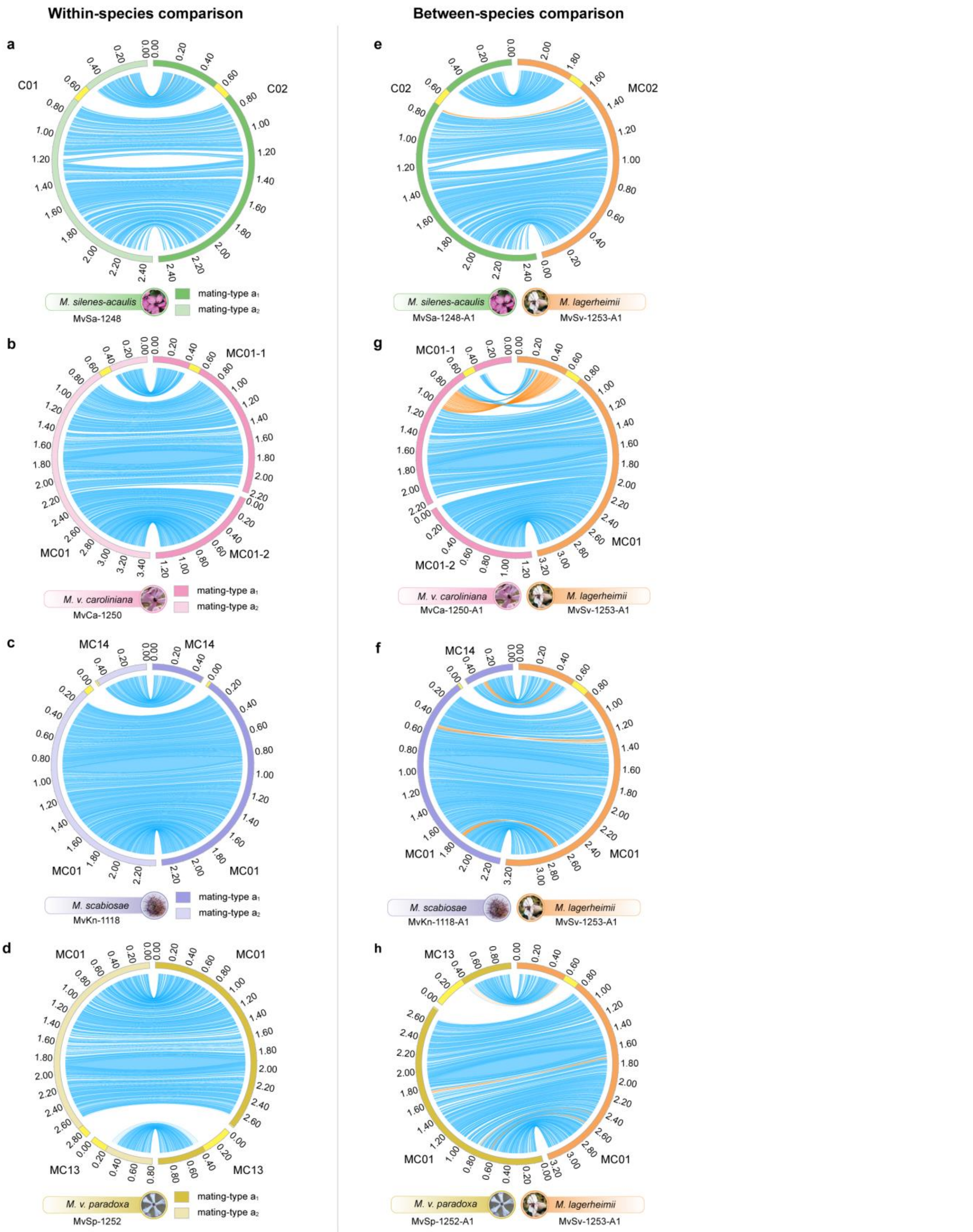

Supplementary Figure 4. Comparison of gene order between autosomes. Blue and orange ribbons connect alleles within-species or single-copy orthologs between-species (i.e. between each focal species and *M. lagerheimii*). The link size is proportional to gene length and orange ribbons represent inversions. Contig size scale is indicated in Megabases. **(a)** to **(h)** Comparisons between the best assembled autosomes within (left) and between (right) species. Yellow regions on the outer track indicate centromeric regions as based on the presence of putative centromere-specific repeats.

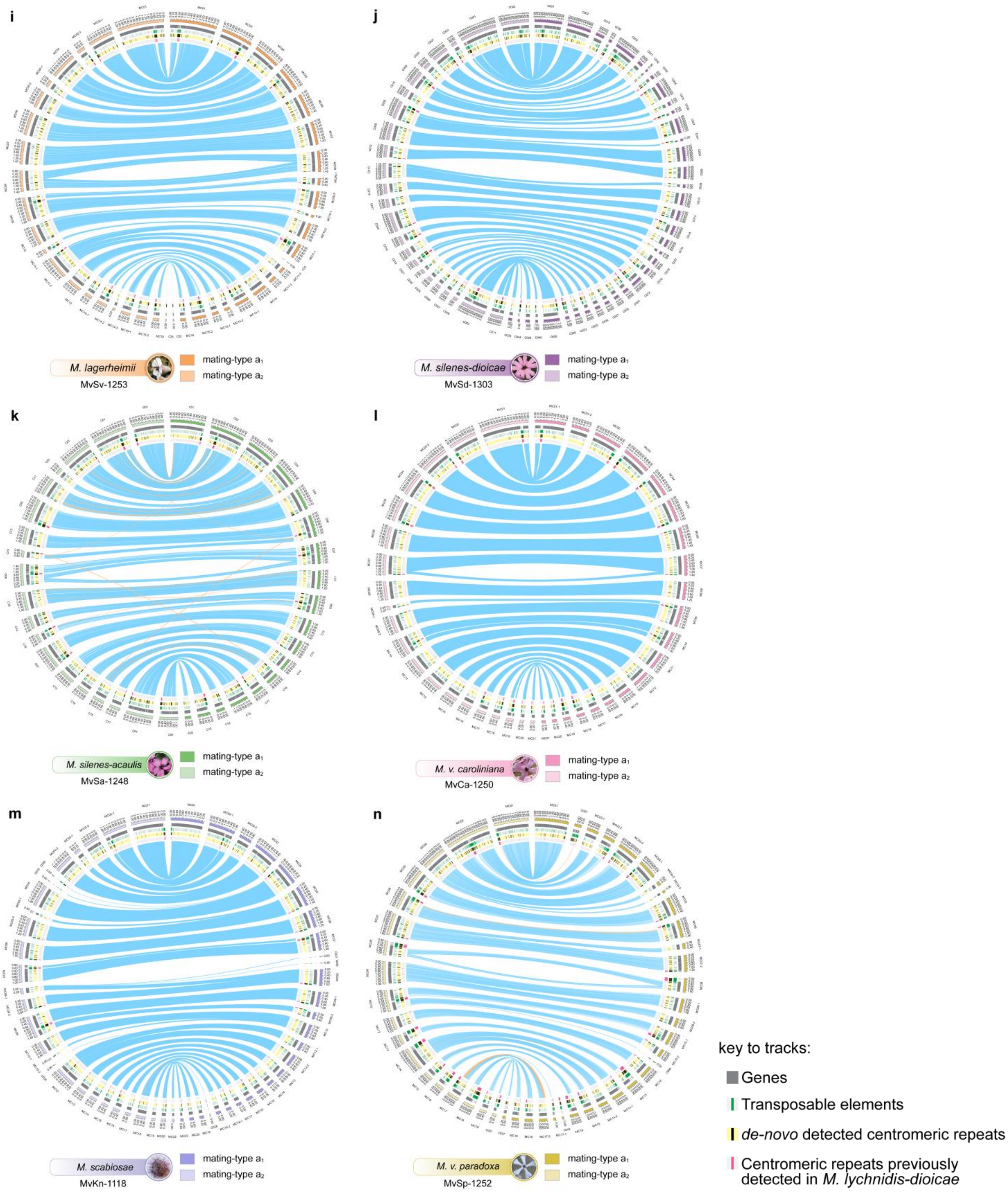

Supplementary Figure 4 (continued). (i) to (n) Within-species comparisons between *Microbotryum* autosomal contigs (a_1 versus a_2 genomes) with all contigs larger than 40 kb represented (except for *M. silenes-acaulis* where only contigs larger than N90 length are plotted due to genome assembly fragmentation). The internal tracks indicate the following features: 1) predicted genes that do not match transposable elements, 2) predicted transposable elements, 3) *M. lagerheimii de-novo* detected centromeric repeats (using the novel method described in the material and methods), and 4) centromeric repeats previously detected in *M. lychnidis-dioicae*[1].

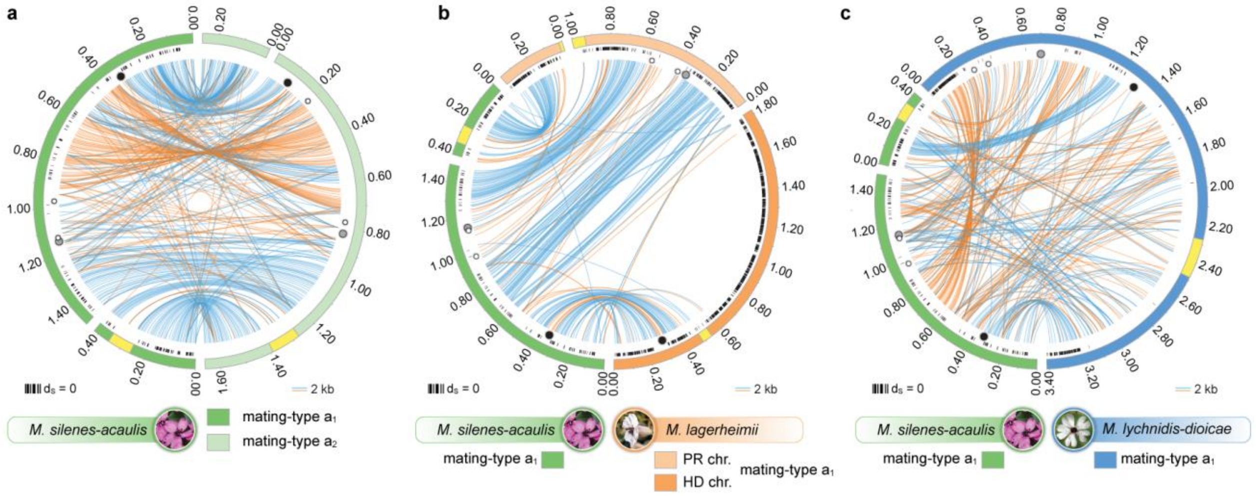

Supplementary Figure 5. Comparison of gene order between mating-type chromosomes in *Microbotryum silenes-acaulis*. The *HD*, *PR* and pheromone genes are indicated by black, grey and white small circles, respectively. Blue and orange lines link single-copy orthologs, the latter corresponding to inversions. Yellow regions on the outer track indicate the putative centromere-specific repeats[1] and black marks along the chromosome/contig track indicate genes that have no synonymous substitutions between a_1 and a_2 alleles within species (d_S=0). Links show contiguous BLASTN similarity across a length (in kb) indicated in each panel. The contig size scale is indicated in Megabases. **(a)** *M. silenes-acaulis* a_2 (left) versus a_1 (right) mating-type chromosomes. The a_1 and a_2 mating-type chromosomes of *M. silenes-acaulis* were assembled in two contigs each. Contig comparison showed they constituted a single chromosome, whose assembly was broken in different locations. The PARs on both edges of the mating-type chromosomes are collinear while the non-recombining region displays a large inversion without significant gene shuffling; **(b)** Comparison between the a_1 mating-type chromosomes of *M. silenes-acaulis* (left) and *M. lagerheimii* (right), taken as a proxy for ancestral gene order[2]. The *M. silenes-acaulis* mating-type chromosome corresponds to the whole *M. lagerheimii PR* chromosome and the small arm of the *M. lagerheimii HD* chromosome. The corresponding mating-type chromosomes in the two species are highly collinear, indicating very recent recombination suppression; **(c)** Comparison between the a_1 mating-type chromosomes of *M. silenes-acaulis* (left) and *M. lychnidis-dioicae* (right). Mating-type chromosomes in the two species appear to be entirely homologous but shared a single PAR (the collinear region close to the *HD* locus). The complete ancestral *PR* chromosome became linked to the same arm of the ancestral *HD* chromosome as in *M. lychnidis-dioicae*, except that the opposite edge of the ancestral *PR* chromosome was juxtaposed to the *HD* chromosome arm. The extremity of the ancestral *PR* chromosome that became a PAR in *M. lychnidis-dioicae* was thus found in the middle of the *M. silenes-acaulis* mating-type chromosomes, and vice-versa, while the two species shared the other PAR (see Fig. 2 for additional details).

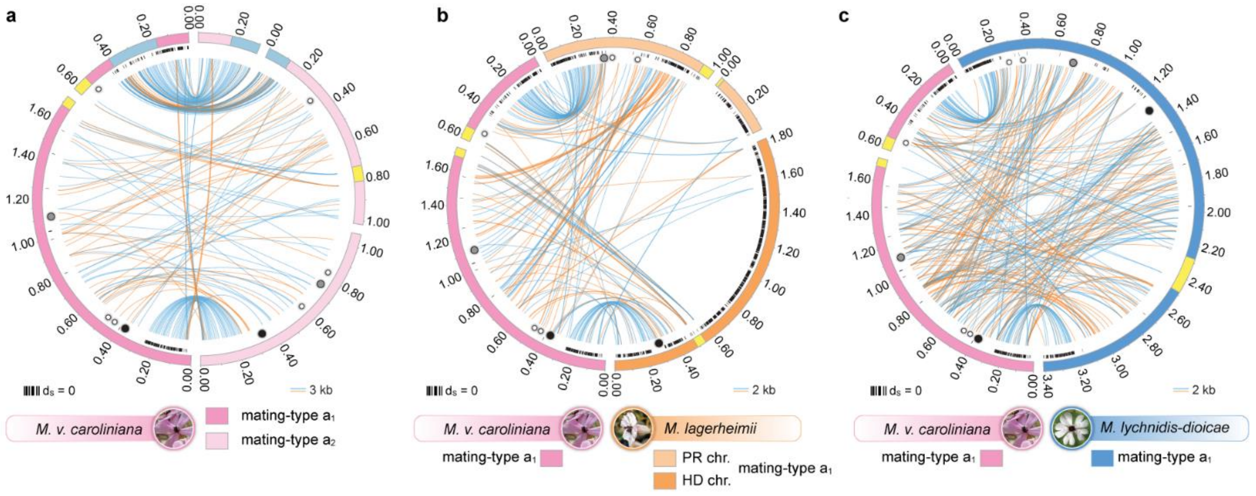

Supplementary Figure 6. Comparison of gene order between mating-type chromosomes in *M. v. caroliniana*. (a) *M. v. caroliniana* a_2 (left) versus a_1 (right) mating-type chromosomes. The a_1 and a_2 mating-type chromosomes of *M. v. caroliniana* were assembled in two contigs each that constitute a single chromosome with assemblies broken in different locations. The two PARs on both edges of the a_1 and a_2 mating-type chromosomes were highly collinear with a_1 - a_2 synonymous substitutions (d_S) close to 0, at the extremities. Conversely, the non-recombining region was large and highly rearranged between the a_1 and a_2 mating-type chromosomes and exhibited non-zero d_S values. The locations of the genes colored in light blue in Fig. 3b are also indicated as light blue boxes on the outer track. **(b)** Comparison between the a_1 mating-type chromosomes of *M. v. caroliniana* (left) and *M. lagerheimii* (right), taken as proxy for the ancestral state[2]. Contrary to the other species where the complete ancestral *PR* chromosome was incorporated as part of the extant mating-type chromosomes, only one arm of each of the *M. lagerheimii PR* and *HD* chromosomes matched the extant *M. v. caroliniana* mating-type chromosome. **(c)** Comparison between a_1 mating-type chromosomes of *M. v. caroliniana* (left) and *M. lychnidis-dioicae* (right). The mating-type chromosome of the two species were mostly homologous and shared two PARs (collinear regions). Contig size scale is indicated in Megabases. All other symbols and features are as described in Supplementary Figure 5.

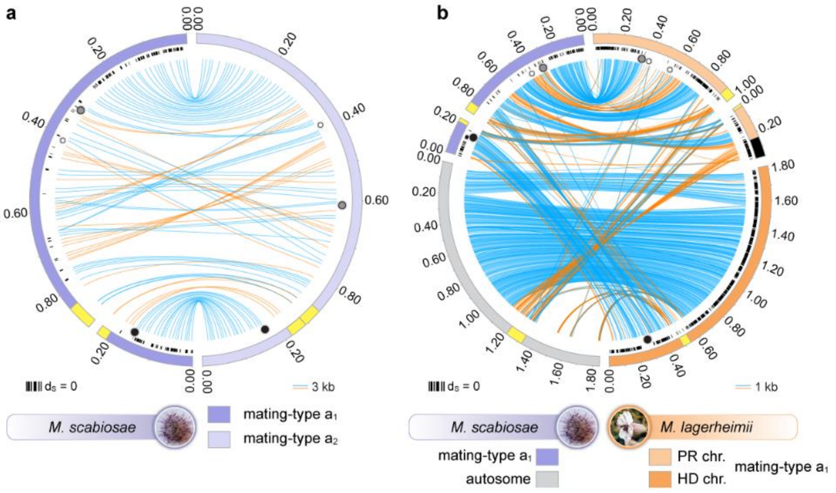

Supplementary Figure 7. Comparison of gene order between mating-type chromosomes in *M. scabiosae*. (a) *M. scabiosae* a_2 (left) versus a_1 (right) mating-type chromosomes. The a_1 and a_2 mating-type chromosomes of *M. scabiosae* were assembled into one and two contigs, respectively, thus constituting a single chromosome. Whereas the PARs on both edges of mating-type chromosomes were collinear and included many zero d_S values, the large non-recombining region had non-zero dS values between a_1 and a_2 mating-types. **(b)** Comparison between the a_1 mating-type chromosome and an autosome of *M. scabiosae* (left) versus a_1 mating-type chromosomes of *M. lagerheimii* (right). The *M. scabiosae* mating-type chromosome corresponds to the *M. lagerheimii PR* chromosome plus the small arm of the *HD* chromosome, resembling patterns in other species. However, the edge of the *M. lagerheimii PR* chromosome (black box on the outer track) corresponded to the center of an *M. scabiosae* autosome suggesting rearrangement within a chromosome arm rather than rearrangement at putative centromeres. **(c)** Comparison between the a_1 mating-type chromosomes of *M. scabiosae* (left) and *M. lychnidis-dioicae* (right). Contig size scale is indicated in Megabases. Symbols and features are as described in Supplementary Figure 5.

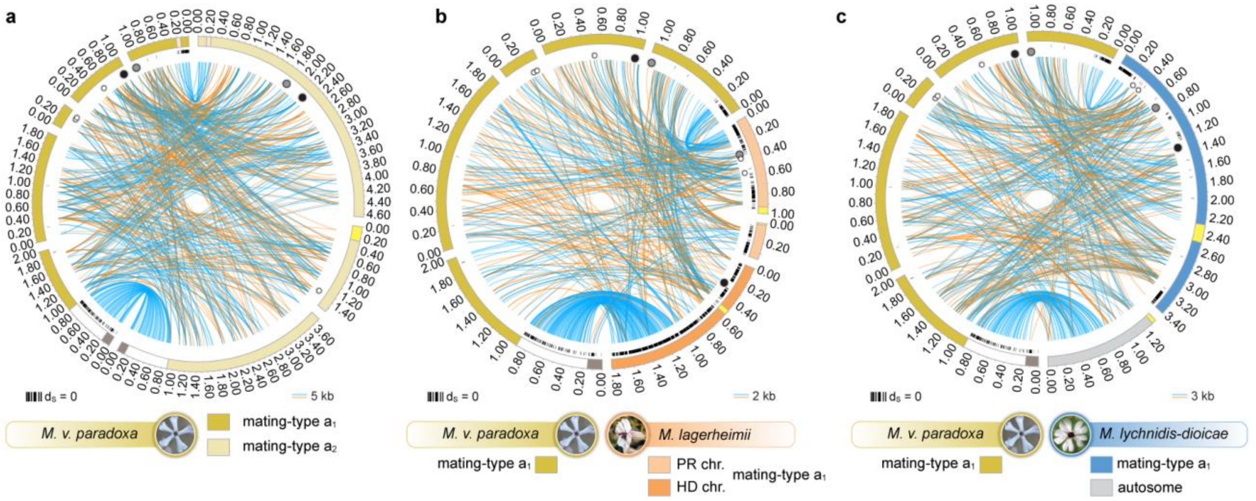

Supplementary Figure 8. Comparison of gene order between mating-type chromosomes in *M. v. paradoxa*. (a) *M. v. paradoxa* a_1 (left) versus a_2 (right) mating-type chromosomes. Some contigs within the non-recombining regions could not be oriented with certainty given the high degree of rearrangements. The locations of the genes colored in pink and white in Fig. 3d are also indicated as pink or white boxes on the outer track. **(b)** Comparison between the a_1 mating-type chromosomes of *M. v. paradoxa* (left) and *M. lagerheimii* (right). Unlike all the other *Microbotryum* species analyzed, where only one arm of the ancestral *HD* chromosome became integrated in the mating-type chromosome, here the whole *HD* and *PR* ancestral chromosomes fused to form the *M. v. paradoxa* mating-type chromosome. An extremity of the *M. v. paradoxa* mating type chromosome (grey region in the outer track) corresponds to rearrangements of regions in the middle of the *M. lagerheimii* mating type chromosomes (see Fig. 2e), supporting complete recombination suppression up to the edge of the *M. v. paradoxa* mating-type chromosome. **(c)** Comparison between the a_1 mating-type chromosome of *M. v. paradoxa* (left) versus a_1 mating-type chromosome and an autosome of *M. lychnidis-dioicae* (right). The mating-type chromosomes in both species were mostly homologous and shared one PAR. The other *M. v. paradoxa* PAR corresponded to the MC12 *M. lychnidis-dioicae* autosome. The grey extremity of the *M. v. paradoxa* mating type chromosome corresponds to rearrangements of regions in the middle of the *M. lychnidis-dioicae* mating type chromosome (Fig. 2e), further supporting complete recombination suppression up to the edge of the *M. v. paradoxa* mating type chromosome. Contig size scale is indicated in Megabases. All other symbols and features are as described in Supplementary Figure 5.

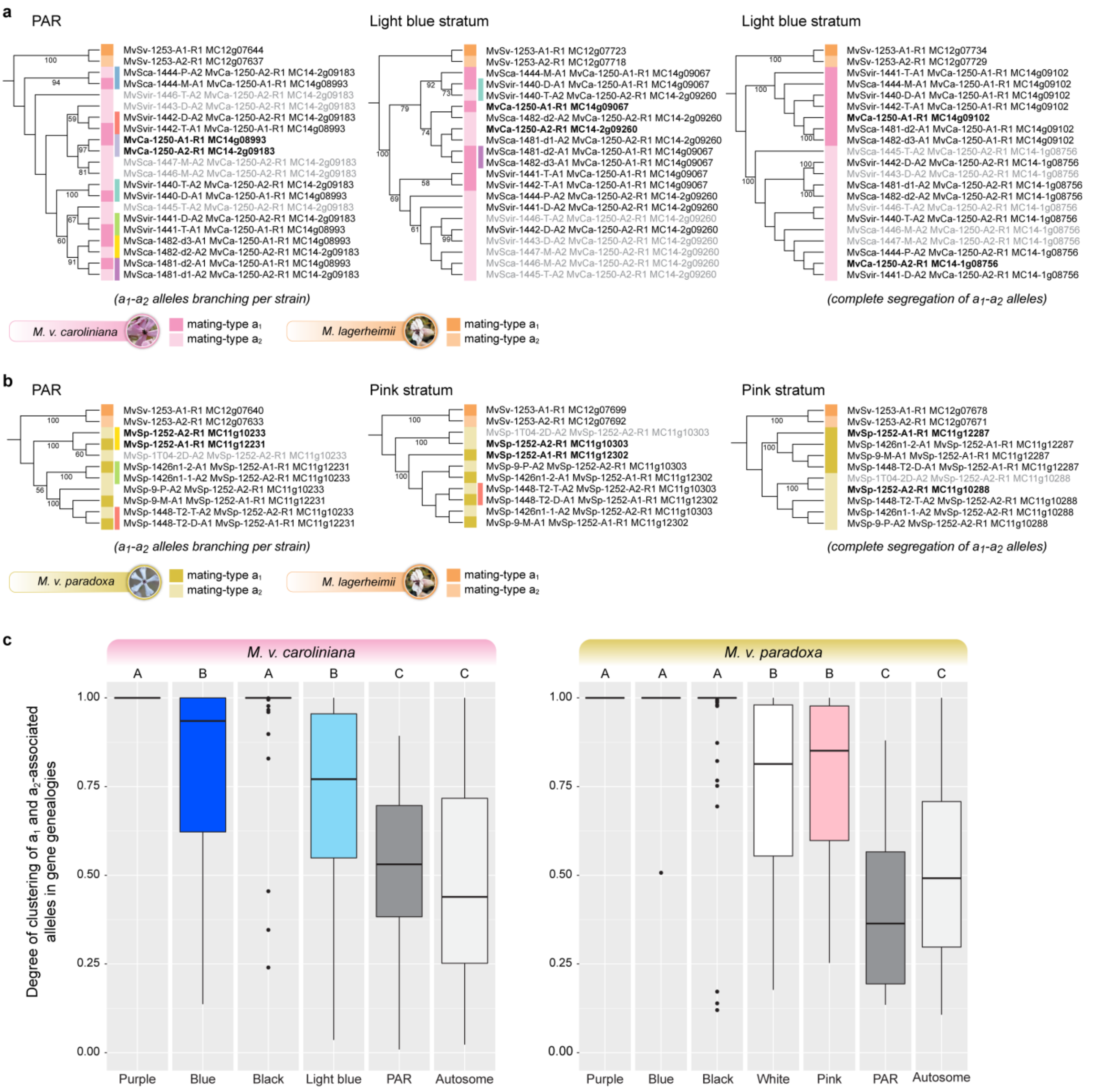

Supplementary Figure 9. Clustering of a_1 versus a_2-associated alleles in 11 strains of *Microbotryum violaceum caroliniana* and 5 strains of *M. v. paradoxa*. Examples of genes genealogies with different levels of clustering of a_1 versus a_2-associated alleles in *M. v. caroliniana* **(a)** and in *M. v. paradoxa* **(b)**; grey-colored sequence names represent strains for which only one mating type was sequenced; sequences in bold are from the reference genomes; a_1 and a_2-associated alleles of the same strain are colored only when clustered in the tree, different diploids having different colors. **(c)** Distribution of the degree of clustering of a_1 versus a_2-associated alleles in *M. v. caroliniana* (left) and *M. v. paradoxa* (right), in different genomic regions (an autosome as well as the various evolutionary strata and the pseudo-autosomal regions (PARs) in mating-type chromosomes). An index of 1 means that a_1 and a_2-associated alleles are fully separated in gene genealogies (see Material and Methods). Different capital letters at the top indicate significantly different means (Student's t-tests, Supplementary Table 4). Number of genes analyzed in *M. v. caroliniana* in the different regions: autosome 529, blue stratum 22, purple stratum 10, black stratum 70, light blue stratum 41, PARs 56. Number of genes analyzed in *M. v. paradoxa* in the different regions: autosome 490, blue stratum 11, purple stratum 11, black stratum 150, pink stratum 24, white stratum 206, PARs 19. The boxplots represent the median (center line), the 25th percentile and 75th percentiles (box bounds), the 5th percentile and 95th percentiles (whiskers), and points being the outliers from these 95th and 5th percentiles.

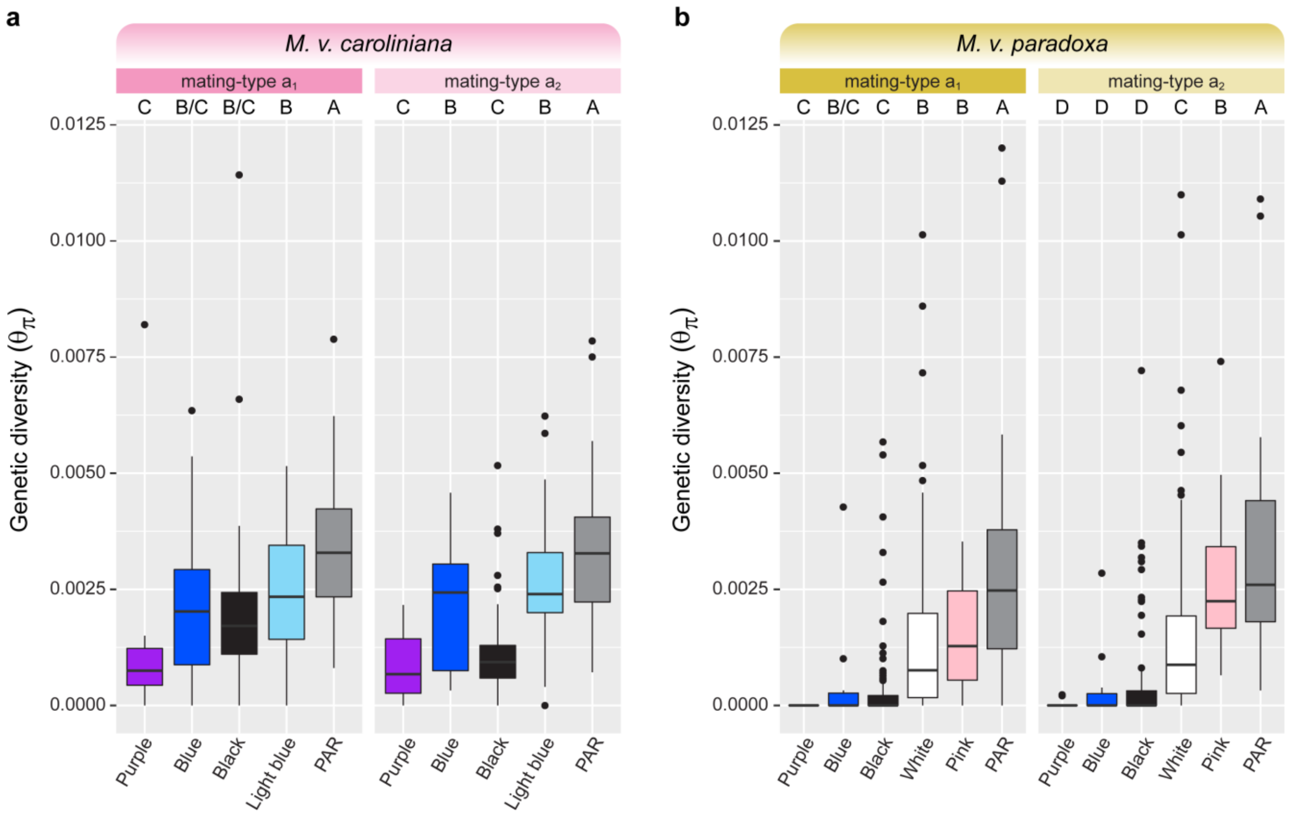

Supplementary Figure 10. Genetic diversity (θπ) in (a) 11 strains of *Microbotryum violaceum caroliniana* and (b) 5 strains of *M. v. paradoxa*. Genetic diversity was computed per species and per mating type, in the different genomic regions of mating-type chromosomes (a_1 and a_2): the old shared evolutionary strata (blue and purple), the young species-specific evolutionary strata (black, white, pink and light blue) and the pseudo-autosomal regions (PARs). Different capital letters at the top indicate significantly different means within species for a given mating type (Student's t-tests, Supplementary Table 5). Number of genes analyzed in *M. v. caroliniana* in the different regions: blue stratum 22, purple stratum 10, black stratum 70, light blue stratum 41, PARs 59. Number of genes analyzed in *M. v. paradoxa* in the different regions: blue stratum 11, purple stratum 11, black stratum 150, pink stratum 24, white stratum 206, PARs 19. The boxplots represent the median (center line), the 25th percentile and 75th percentiles (box bounds), the 5th percentile and 95th percentiles (whiskers), the points being the outliers from these 95th and 5th percentiles.

Supplementary Table 1. Statistics on the genomes and mating-type chromosomes of the *Microbotryum* species analyzed in this study, and in the different genomic partitions of the mating-type chromosomes: recombining regions (RR), non-recombining regions (NRR), pseudo-autosomal regions (PAR) for **(a)** *M. lagerheimii* (with unlinked mating type loci; statistics therefore are given for the *HD* and *PR* mating-type chromosomes), **(b)** the remaining species with linked *PR* and *HD* loci, and thus a single mating-type chromosome, and (c) whole genome assembly statistics.

(a)

			M. lagerheimii
No. of mating-type chromosomes			2
No. of contigs for the	*HD*	a_1 chromosome	1
		a_2 chromosome	2
	PR	a_1 chromosome	2
		a_2 chromosome	2
Size (bp) of the	*HD*	a_1 chromosome	1,823,320
		a_2 chromosome	1,828,681
	PR	a_1 chromosome	1,368,423
		a_2 chromosome	1,300,106
Size (bp / %) of the RR on the	*HD*	a_1 chromosome	1,567,057 / 85.95
		a_2 chromosome	1,577,456 / 86.26
	PR	a_1 chromosome	681,586 / 49.81
		a_2 chromosome	659,051 / 50.69
Size (bp / %) of the NRR on the	*HD*	a_1 chromosome	256,263 / 14.05
		a_2 chromosome	251,225 / 13.74
	PR	a_1 chromosome	686,837 / 50.19
		a_2 chromosome	641,055 / 49.31

(b)

		M. scabiosae	***M. silenes-acaulis***	***M. v. caroliniana***	***M. v. paradoxa***
No. of mating-type chromosomes		1	1	1	1
No. of contigs for the mating-type chromosome	a_1	1	2	2	4
	a_2	2	2	3	3
Size (bp) of the mating-type chromosome	a_1	1,129,805	1,909,079	2,353,735	5,963,545
	a_2	1,163,263	1,924,428	2,385,087	9,963,855
Size (bp / %) of the PAR of the mating-type chromosome	a_1	411,060 / 36.4	996,791 / 52.2	407,308 / 17.3	140,099 / 2.18
	a_2	424,491 / 36.5	1,039,328 / 54.0	395,275 / 16.7	146,725 / 1.47
Size (bp / %) of the NRR of the mating-type chromosome	a_1	718,744 / 63.6	912,288 / 47.8	1,701,704 / 72.3	6,294,107 / 97.82
	a_2	738,771 / 63.5	885,100 / 45.9	1,734,165 / 72.7	9,817,130 / 98.53

Supplementary Table 1 (continued).

(c)

**Genome**	**Contig number**	**Length (bp)**	**N50 (bp)**	**Percent masked for repeats**	**Reference**
M. intermedium	24	23,461,035	1,644,950	0.0625	Branco et al. 2017
M. lagerheimii a_1	42	25,806,628	1,584,903	0.0743	Branco et al. 2017
M. lagerheimii a_2	37	25,667,824	1,585,144	0.0764	Branco et al. 2017
M. lychnidis-dioicae a_1	48	29,901,156	1,736,850	0.1778	Branco et al. 2017
M. lychnidis-dioicae a2	37	30,318,316	1,730,088	0.1719	Branco et al. 2017
M. silenes-acaulis a_1	77	29,349,019	1,490,411	0.1410	This study
M. silenes-acaulis a_2	89	29,858,089	1,555,341	0.1454	This study
M. scabiosae a_1	123	24,532,870	1,157,998	0.0811	This study
M. scabiosae a_2	147	25,410,563	1,198,341	0.0797	This study
M. silenes-dioicae a_1	144	33,593,023	936,137	0.2302	Branco et al. 2017
M. silenes-dioicae a_2	128	33,958,966	1,321,747	0.2331	Branco et al. 2017
M. v. caroliniana a_1	131	28,837,994	1,517,213	0.1433	This study
M. v. caroliniana a_2	137	28,906,851	1,311,234	0.1419	This study
M. v. paradoxa a_1	225	40,892,155	1,035,691	0.2212	This study
M. v. paradoxa a_2	156	39,900,209	1,869,174	0.2196	This study
Rhodotorula babjevae a1	29	21,772,635	1,453,553	0.0025	Branco et al. 2017
Rhodotorula babjevae a2	34	21,676,322	1,321,968	0.0038	Branco et al. 2017

Supplementary Table 2. Comparisons of synonymous substitution (d_S) means across genes between evolutionary strata (referred by the colors used in Fig. 3) and the pseudo-autosomal regions (PARs) for *Microbotryum silenes-acaulis*, *M. v. caroliniana*, *M. v. paradoxa* and *M. scabiosae*. Number of genes analyzed in *M. silenes-acaulis* in the different genomic regions: blue and purple strata pooled 20, black stratum 67, pink stratum 24, PARs 118. Number of genes analyzed in *M. scabiosae* in the different regions: blue and purple strata pooled 21, black stratum 66, PARs 69. Number of genes analyzed in *M. v. caroliniana* in the different regions: blue and purple strata pooled 13, black stratum 46, light blue stratum 29, PARs 48. Number of genes analyzed in *M. v. paradoxa* in the different regions: blue and purple strata pooled 8, black stratum 69, pink stratum 17, white stratum 140, PARs 11. **(a)** d_S (mean and standard error) across genes between the large putative strata. **(b)** Analyses of variance (ANOVA) per species. **(c)** Pairwise mean comparisons per species and genomic region (Student's t tests), with statistically significant differences indicated in bold and with an asterisk. The d_S distributions significantly deviated from normality (Shapiro-Wilk tests, W= 0.10, P<00001 for *M. silenes-acaulis*, W= 0.18, P<00001 for *M. v. caroliniana*, W= 0.41, P<00001, for *M. v. paradoxa,* W= 0.18, P<00001 for *M. scabiosae*) and variances were significantly different among genomic regions for some species (Levene tests, F-ratio= 1.00, d.f.=3, P=0.41 for *M. silenes-acaulis*, F-ratio= 2.80, d.f.=4, P=0.028 for *M. v. caroliniana*, F-ratio= 67.09, d.f.=4, P<0.0001 for *M. v. paradoxa,* F-ratio= 4.23, d.f.=4, P=0.01 for *M. scabiosae*). **(d)** Kruskal-Wallis non-parametric tests**,** also showing significant differences among genomic regions.

(a)

**Stratum**	Mean d_S ± SE			
	M. silenes-acaulis	***M. v. caroliniana***	***M. scabiosae***	***M. v. paradoxa***
Blue and purple strata	0.241±0.149	0.092±0.069	0.090±0.044	0.299±0.365
Black stratum	0.003±0.001	0.102±0.050	0.048±0.013	0.099±0.001
Light blue stratum		0.005±0.001		
Pink stratum				0.012±0.002
White stratum				0.005±0.000
PARs	0.003±0.001	<0.0001	0.051±0.046	<0.0001

(b)

Species	d.f.	Sum of squares	F-ratio	*P-value*
M. silenes-acaulis	2	1.112	10.94	**<0.0001***
M. v. caroliniana	3	0.323	2.3659	0.0739
M. scabiosae	2	0.030	0.20	0.8182
M. v. paradoxa	4	0.860	36.84	**<0.0001***

Supplementary Table 2. Continued.

(c)

M. silenes-acaulis					
Stratum 1	Stratum 2	Mean difference	Standard error	Student's t	*P*-value
**Blue and purple**	**Black**	**0.2385**	**0.0556**	**4.28**	**<0.0001***
**Blue and purple**	**PAR**	**0.2378**	**0.0523**	**4.54**	**<0.0001***
PAR	Black	0.0004	0.0348	0.01	0.9900
M. v. caroliniana					
Stratum 1	Stratum 2	Mean difference	Standard error	Student's t	*P* -value
**Black**	**PAR**	**0.1025**	**0.0440**	**2.33**	**0.0215***
Black	Light blue	0.0973	0.0506	1.92	0.0567
Blue and purple	PAR	0.0916	0.0667	1.37	0.1720
Blue and purple	Light blue	0.0865	0.0712	1.21	0.2271
Black	Blue and purple	0.0108	0.0670	0.16	0.8720
Light blue	PAR	0.0052	0.0502	0.10	0.9178
M. scabiosae (degrees of freedom = 153)					
Stratum 1	Stratum 2	Mean difference	Standard error	Student's t	*P* -value
Blue and purple	Black	0.04216	0.0687	0.61	0.5405
Blue and purple	PAR	0.03906	0.0684	0.57	0.5686
PAR	Black	0.00310	0.0472	0.06	0.9478
M. v. paradoxa (degrees of freedom = 240)					
Stratum 1	Stratum 2	Mean difference	Standard error	Student's t	*P* -value
**Blue and purple**	**PAR**	**0.2690**	**0.0355**	**7.58**	**<0.0001***
**Blue and purple**	**White**	**0.2635**	**0.0277**	**9.49**	**<0.0001***
**Blue and purple**	**Pink**	**0.2569**	**0.0327**	**7.84**	**<0.0001***
**Blue and purple**	**Black**	**0.1696**	**0.0285**	**5.94**	**<0.0001***
**Black**	**PAR**	**0.0994**	**0.0248**	**4.01**	**<0.0001***
**Black**	**White**	**0.0939**	**0.0112**	**8.35**	**<0.0001***
**Black**	**Pink**	**0.0873**	**0.0207**	**4.22**	**<0.0001***
Pink	PAR	0.0121	0.0296	0.41	0.6594
Pink	White	0.0066	0.0196	0.34	0.7684
White	PAR	0.0055	0.0239	0.23	0.3679

(d)

Species	d.f.	Chi2	*P-value*
M. silenes-acaulis	2	5.69	P=0.0581
M. v. caroliniana	3	65.54	**P<0.0001***
M. scabiosae	2	22.22	**P<0.0001***
M. v. paradoxa	4	129.26	, **P<0.0001***

Supplementary Table 3. Strains used for polymorphism analyses: strain ID, *Microbotryum* species and its abbreviation, host species of collection, location of collection, mating type sequenced and coverage of sequencing.

strain ID	*Microbotryum* species and abbreviation	Host species	Location of collection	GPS coordinates	Mating types	Sequencing coverage
1T04	*M. violaceum paradoxa*	*S. paradoxa*	Volpaie, near Lamole, Italy	N42 26.298' E13 34.584	a2	20x
1426	*M. violaceum paradoxa*	*S. paradoxa*	Volpaie, near Lamole, Italy	N42 26.298' E13 34.584	a1 & a2	38x 26x
1448	*M. violaceum paradoxa*	*S. paradoxa*	Volpaie, near Lamole, Italy	N42 26.298' E13 34.584	a1 & a2	50x 42x
9	*M. violaceum paradoxa*	*S. paradoxa*	Volpaie, near Lamole, Italy	43°32'35.7"N 11°21'35.1"E"	a1 & a2	38x 34x
1440	*M. violaceum caroliniana*	*S. caroliniana*	Cliftons Pond, North Carolina	35°59'58.2"N 78°20'51.2"W	a1 & a2	68x 46x
1441	*M. violaceum caroliniana*	*S. virginica*	Route 8, Floyd County, Virginia, US	36°50'35.8"N 80°19'00.1"W	a1 & a2	56x 60x
1442	*M. violaceum caroliniana*	*S. virginica*	Charlottesville Reservoir, Virginia, US	38°01'35.5"N 78°33'31.8"W	a1 & a2	48x 56x
1443	*M. violaceum caroliniana*	*S. virginica*	Charlottesville Reservoir, Virginia, US	38°01'35.5"N 78°33'31.8"W	a2	38x
1444	*M. violaceum caroliniana*	*S. caroliniana*	Blue Ridge Parkway, US	37°44'46.5"N 79°17'59.7"W	a1 & a2	66x 46x
1445	*M. violaceum caroliniana*	*S. caroliniana*	Gilbert Creek, Kentucky, US	37°58'28.9"N 84°50'46.0"W	a2	50x
1446	*M. violaceum caroliniana*	*S. virginica*	Old Garth Road, Charlottesville, Virginia, US	38°03'38.6"N 78°31'57.5"W	a2	56x
1446	*M. violaceum caroliniana*	*S. virginica*	Old Garth Road, Charlottesville, Virginia, US	38°03'38.6"N 78°31'57.5"W	a2	50x
1447	*M. violaceum caroliniana*	*S. virginica*	Sugar Hollow, near Charlottesville, Virginia, US	38°08'16.8"N 78°44'23.3"W	a2	54x
1481	*M. violaceum caroliniana*	*S. caroliniana*	Site 2 Virginia near Briary gap route 257, US	38°25'44.8"N 79°02'09.9"W	a1 & a2	52x 40x
1482	*M. violaceum caroliniana*	*S. caroliniana*	Site 2 Virginia near Briary gap route 257, US	38°25'44.8"N 79°02'09.9"W	a1 & a2	38x

Supplementary Table 4. Comparisons of the degree of clustering of a_1 versus a_2-associated alleles in multiple genomes of *Microbotryum violaceum caroliniana* (a) and *M. v. paradoxa* (b) using Student's t tests, pairwise among the different genomic regions of mating-type chromosomes: the old shared evolutionary strata (blue and purple), the young species-specific evolutionary strata (black, white, pink and light blue) and the pseudo-autosomal regions (PARs). ANOVAs were significant for *M. v. caroliniana* (Sum of squares 3.68, d.f. 5, F-ratio 50.86, P<0.0001) and for *M. v. paradoxa* (Sum of squares 30.95, d.f. 6, F-ratio 91.09, P<0.0001). The index distributions significantly deviated from normality (Shapiro-Wilk tests, W= 0.92 for *M. v. caroliniana*, W= 0.89 for *M. v. paradoxa*; P<00001 for both) and variances were significantly different among genomic regions (Levene tests, F-ratio= 28.31, d.f.=5, P<0.0001 for *M. v. caroliniana*; F-ratio= 41.27, d.f.=6, P<0.0001 for *M. v. paradoxa*). However, non-parametric tests also showed significant differences among genomic regions (Kruskal-Wallis tests, Chi2= 211.35, d.f.=5, P<0.0001 for *M. v. caroliniana*; Chi2= 389.97, d.f.=6, P<0.0001 for *M. v. paradoxa*). Number of genes analyzed in *M. v. caroliniana* in the different regions: autosome 529, blue stratum 22, purple stratum 10, black stratum 70, light blue stratum 41, PARs 56. Number of genes analyzed in *M. v. paradoxa* in the different regions: autosome 490, blue stratum 11, purple stratum 11, black stratum 150, pink stratum 24, white stratum 206, PARs 19.

**(a) *M. v. caroliniana***				
Stratum 1	Stratum 2	Difference	Student's t	*P* -value
**purple**	**Autosome**	**0.5168507**	**6.02**	**<.0001***
**black**	**Autosome**	**0.4833078**	**14.12**	**<.0001***
**purple**	**PAR**	**0.4744464**	**5.14**	**<.0001***
**black**	**PAR**	**0.4409036**	**-9.14**	**<.0001***
**purple**	**Light blue**	**0.3092683**	**3.26**	**0.0012***
**blue**	**Autosome**	**0.2921234**	**4.99**	**<.0001***
**black**	**Light blue**	**0.2757254**	**-5.21**	**<.0001***
**blue**	**PAR**	**0.2497192**	**-3.69**	**0.0002***
**purple**	**blue**	**0.2247273**	**2.19**	**0.0289***
**Light blue**	**Autosome**	**0.2075824**	**4.76**	**<.0001***
**black**	**blue**	**0.1911844**	**-2.91**	**0.0038***
**Light blue**	**PAR**	**0.1651781**	**-2.99**	**0.0029***
blue	Light blue	0.0845410	-1.19	0.2349
PAR	Autosome	0.0424042	1.12	0.2625
purple	black	0.0335429	0.37	0.7124

Supplementary Table 4. Continued.

**(b)** ***M. v. paradoxa***				
Stratum 1	Stratum 2	Difference	Student's t	*P* -value
**purple**	**PAR**	**0.5935789**	**6.58**	**<.0001***
**black**	**PAR**	**0.5684323**	**-9.8**	**<.0001***
**blue**	**PAR**	**0.5487608**	**-6.09**	**<.0001***
**purple**	**Autosome**	**0.4886918**	**6.73**	**<.0001***
**black**	**Autosome**	**0.4635452**	**20.87**	**<.0001***
**blue**	**Autosome**	**0.4438737**	**6.12**	**<.0001***
**pink**	**PAR**	**0.3590373**	**4.91**	**<.0001***
**white**	**PAR**	**0.3400984**	**5.96**	**<.0001***
**pink**	**Autosome**	**0.2541502**	**5.11**	**<.0001***
**purple**	**white**	**0.2534806**	**-3.44**	**0.0006***
**white**	**Autosome**	**0.2352113**	**11.90**	**<.0001***
**purple**	**pink**	**0.2345417**	**2.71**	**0.0069***
**black**	**white**	**0.2283339**	**-8.94**	**<.0001***
**black**	**pink**	**0.2093950**	**-4.01**	**<.0001***
**blue**	**white**	**0.2086624**	**-2.83**	**0.0047***
**blue**	**pink**	**0.1897235**	**-2.19**	**0.0288***
Autosome	PAR	0.1048871	-1.88	0.0598
purple	blue	0.0448182	0.44	0.6588
purple	black	0.0251467	0.34	0.7352
black	blue	0.0196715	-0.26	0.7914
pink	white	0.0189389	-0.37	0.7122

Supplementary Table 5. Comparisons of the genetic diversity ($\theta\pi$) among genomic regions in multiple genomes of (a) *Microbotryum violaceum caroliniana* a_1 (b) *M. v. caroliniana* a_2 (c) *M. v. paradoxa* a_1 and (d) *M. v. paradoxa* a_2 using Student's t tests, comparing the different genomic regions of mating-type chromosomes (a_1 and a_2): the old shared evolutionary strata (blue and purple), the young species-specific evolutionary strata (black, white, pink and light blue) and the pseudo-autosomal regions (PARs). ANOVAs were significant for *M. v. caroliniana* a_1 (sum of squares 0.000075, d.f. 4, F-ratio 8.37, P<0.0001), *M. v. caroliniana* a_2 (sum of squares 0.000181, d.f. 4, F-ratio 29.01, P<0.0001), *M. v. paradoxa* a_1 (sum of squares 0.00021, d.f. 5, F-ratio 21.57, P<0.0001) and *M. v. paradoxa* a_2 (sum of squares 0.00028, d.f. 5, F-ratio 24.47, P<0.0001). The genetic diversity ($\theta\pi$) distributions significantly deviated from normality (Shapiro-Wilk tests, W= 0.90, P<0.00001 for *M. v. caroliniana* a_1, W= 0.93, P<0.0001 for *M. v. caroliniana* a_2, W= 0.65, P<0.00001 for *M. v. paradoxa* a_1, = 0.69, P<0.00001 for *M. v. paradoxa* a_2) and variances were significantly different among genomic regions (Levene tests, F-ratio=7.96, d.f.=4, P<0.0001 for *M. v. caroliniana* a_1; F-ratio=37.78, d.f.=4, P<0.0001 for *M. v. caroliniana* a_2; F-ratio=43.31, d.f.=5, P<0.0001 for *M. v. paradoxa* a_2). However, non-parametric tests also showed significant differences among genomic regions (Kruskal-Wallis tests, Chi2= 48.32, d.f.=4, P<0.0001 for *M. v. caroliniana* a_1; Chi2= 82.97, d.f.=4, P<0.0001 for *M. v. caroliniana* a_2; Chi2= 130.32, d.f.=5, P<0.0001 for *M. v. paradoxa* a_1; Chi2= 149.58, d.f.=5, P<0.0001 for *M. v. paradoxa* a_2). Number of genes analyzed in *M. v. caroliniana* in the different regions: blue stratum 22, purple stratum 10, black stratum 70, light blue stratum 41, PARs 59. Number of genes analyzed in *M. v. paradoxa* in the different regions: blue stratum 11, purple stratum 11, black stratum 150, pink stratum 24, white stratum 206, PARs 19.

**(a) *M. v. caroliniana a₁***				
Stratum 1	Stratum 2	Difference	Student's t	*P* -value
**PAR**	**purple**	**0.0019131**	**-3.74**	**0.0002***
**PAR**	**black**	**0.0013819**	**5.23**	**<.0001***
**PAR**	**blue**	**0.0010881**	**2.91**	**0.0040***
**Light blue**	**purple**	**0.0010654**	**-2.02**	**0.0448***
**PAR**	**Light blue**	**0.0008476**	**2.79**	**0.0058***
blue	purple	0.0008249	-1.45	0.1498
Light blue	black	0.0005343	1.81	0.0709
black	purple	0.0005311	-1.05	0.2949
blue	black	0.0002938	0.80	0.4226
Light blue	blue	0.0002405	0.61	0.5437

**(b) *M. v. caroliniana a₂***				
Stratum 1	Stratum 2	Difference	Student's t	*P* -value
**PAR**	**purple**	**0.0024554**	**-5.75**	**<.0001***
**PAR**	**black**	**0.0021985**	**9.96**	**<.0001***
**Light blue**	**purple**	**0.0017719**	**-4.02**	**<.0001***
**Light blue**	**black**	**0.0015151**	**6.17**	**<.0001***
**blue**	**purple**	**0.0013512**	**-2.84**	**0.0050***
**PAR**	**blue**	**0.0011042**	**3.54**	**0.0005***
**blue**	**black**	**0.0010943**	**3.58**	**0.0004***
**PAR**	**Light blue**	**0.0006834**	**2.69**	**0.0077***
Light blue	blue	0.0004208	**1.27**	0.2038
black	purple	0.0002568	**-0.61**	0.5436

Supplementary Table 5. Continued.

**(c)** ***M. v. paradoxa a₁***				
Stratum 1	Stratum 2	Difference	Student's t	*P* -value
**PAR**	**purple**	**0.0032774**	**-6.35**	**<.0001***
**PAR**	**black**	**0.0030036**	**9.01**	**<.0001***
**PAR**	**blue**	**0.0027362**	**5.68**	**<.0001***
**PAR**	**white**	**0.0020227**	**-6.23**	**<.0001***
**PAR**	**pink**	**0.0017545**	**-2.24**	**<.0001***
**pink**	**purple**	**0.0015229**	**-4.72**	**0.0029***
**white**	**purple**	**0.0012547**	**2.95**	**0.0039***
**pink**	**black**	**0.0012491**	**6.85**	**<.0001***
pink	blue	0.0009818	**4.02**	0.0541
**white**	**black**	**0.0009809**	**6.52**	**<.0001***
white	blue	0.0007136	2.13	0.0994
blue	purple	0.0005411	-0.60	0.3639
black	purple	0.0002738	-0.68	0.5305
pink	white	0.0002682	-3.74	0.3736
blue	black	0.0002674	0.13	0.5402

**(d)** ***M. v. paradoxa a₂***				
Stratum 1	Stratum 2	Difference	Student's t	*P* -value
**PAR**	**purple**	**0.0035103**	**-6.20**	**<.0001***
**PAR**	**black**	**0.0032006**	**8.83**	**<.0001***
**PAR**	**blue**	**0.0031398**	**5.17**	**<.0001***
**pink**	**purple**	**0.0025074**	**-2.99**	**<.0001***
**pink**	**black**	**0.0021978**	**4.07**	**<.0001***
**PAR**	**white**	**0.0021792**	**-6.04**	**<.0001***
**pink**	**blue**	**0.0021370**	**1.93**	**<.0001***
**white**	**purple**	**0.0013311**	**2.90**	**0.0034***
**pink**	**white**	**0.0011764**	**-0.89**	**0.0002***
**white**	**black**	**0.0010214**	**6.55**	**<.0001***
**PAR**	**pink**	**0.0010028**	**-4.09**	**0.0257***
**white**	**blue**	**0.0009606**	**1.65**	**0.0339***
blue	purple	0.0003705	**-0.91**	0.5516
black	purple	0.0003097	**-0.63**	0.4970
blue	black	0.0000608	**0.61**	0.8939

Supplementary Table 6. Analyses of variance (ANOVA) testing the effect of species, genomic region (autosomes, PARs, and the different evolutionary strata) and mating-type (a_1 vs. a_2). (a) Transposable element proportions in six species, two mating types and 10 genomic regions (an autosome, pseudo-autosomal regions, and the 12 evolutionary strata across species that could be delimited in the current state and used for TE count). The TE content distribution significantly deviated from normality (Shapiro-Wilk tests, W= 0.74 P<0001) and variances were significantly different among genomic regions (Levene test, F-ratio= 4.55, d.f.=11, P<0.0001). However, a non-parametric test also showed significant differences among genomic regions (Kruskal-Wallis test, Chi2= 27.25, d.f.=11, P=0.004). **(b) Gene loss proportions** in six species, two mating types and eight genomic regions (an autosome, pseudo-autosomal regions, and the seven evolutionary strata across species that could be delimited in the current state and for which gene losses could be assessed)**.** Statistically significant results are indicated in bold and with an asterisk. The gene loss distribution significantly deviated from normality (Shapiro-Wilk tests, W= 0.77, P<0001) and variances were significantly different among genomic regions (Levene test, F-ratio= 11.70, d.f.=7, P<0.0001). However, a non-parametric test also showed significant differences among genomic regions (Kruskal-Wallis test, Chi2= 40.55, d.f.=7, P<0.0001).

(a)

Source	DF	Sum of squares	F ratio	P-value
Genomic regions	11	0.0533	94000	**<0.0001***
Species	5	0.0084	3.271	**0.0170***
Mating type	1	<0.001	0.078	0.7822

(b)

Source	DF	Sum of squares	F ratio	P-value
Genomic regions	6	1.3700	22.397	**<0.0001***
Species	5	0.2346	4.602	**0.0027***
Mating type	1	0.0010	0.099	0.7552

Supplementary Note 1: Script for the computation of the clustering index in gene genealogies

```
##
## Rscript for paper 'Multiple convergent supergene evolution events in mating-type chromosomes'
## Branco et al., In revision.
##

require(ape)
## this function takes as input the nodal distance matrix of a tree and returns
## an array obtained by successively sampling (randomly in the case of multiple identical distances)
## the minimum distance between A1s and A2s.
minvals<-function(m) {
        A1<-grep("A1",colnames(m))
        A2<-grep("A2",colnames(m))
        M<-melt(m[A1,A2])
        cpt<-0
        val<-array()
        while(nrow(M)>1) {
                cpt<-cpt+1
                MiNis<-which(M[,3]==min(M[,3]))
                if (length(MiNis)==1) mini<-MiNis
                else mini<-sample(MiNis,1)
                val[cpt]<-M[mini,3]
                names<-c(as.character(M[mini,1]), as.character(M[mini,2]))
                M<-M[!is.element(M[,1],names)&!is.element(M[,2],names),]
        }
        return(val)
}



##for a given tree 'tr', this function
##returns the index described in the M&M.
ComputeScore<-function(tr) {
        tr<-drop.tip(tr, tr$tip.label[grep("^MvSv-", tr$tip.label)]) ##remove outgroup
        tr<-compute.brlen(tr,1) ##transform patristic to nodal distance (we only use topology for the
test)
        tr$edge.length[is.element(tr$edge[,1],which(as.numeric(tr$node.label)<30)+Ntip(tr))&tr$edge[,2
]>Ntip(tr)]<-0 ##Suppress badly supported nodes (bootstrap < 30)
        tr<-di2multi(tr)
        tr<-compute.brlen(tr,1)
        mat<-cophenetic(tr) ##compute pairwise distance matrix

        meandist1<-mean(replicate(10,mean(minvals(mat)))) #replicate 10 times the computation of the
observed score with mincals (see M&M)
        meanperm<-array()
        for (i in 1:1000) {
                #reshuffling of the matrix
                new<-sample(colnames(mat))
                colnames(mat)<-new
                rownames(mat)<-new
                mat<-mat[new,new]
                #computation of the score after reshuffling
                meanperm[i]<-mean(minvals(mat))
        }
        res<-sum(meandist1>=meanperm)/1000 ##non parametric test: proportion of the reshuffling giving
smaller value of the computed score
        res
}
```

Supplementary References

1 Badouin, H., Hood, M. E., Gouzy, J., Aguileta, G., Siguenza, S., Perlin, M. H., Cuomo, C. A., Fairhead, C., Branca, A. & Giraud, T. Chaos of rearrangements in the mating-type chromosomes of the anther-smut fungus *Microbotryum lychnidis-dioicae*. *Genetics* **200**, 1275-1284 (2015).

2 Branco, S., Badouin, H., Rodríguez de la Vega , R., Gouzy, J., Carpentier, F., Aguileta, G., Siguenza, S., Brandenburg, J., Coelho, M., Hood, M. & Giraud, T. Evolutionary strata on young mating-type chromosomes despite lack of sexual antagonism. *Proceedings of the National Academy of Sciences of the United States of America* **114**, 7067-7072 (2017).
